# Near-unity fueling light into a single plasmonic nanocavity

**DOI:** 10.1515/nanoph-2025-0331

**Published:** 2025-11-06

**Authors:** Haiming Ye, Junhao Ge, Zhengyi Lu, Dudu Song, Jiamin Ji, Zhaoyang Peng, Shunping Zhang, Hongxing Xu

**Affiliations:** Key Laboratory of Artificial Micro- and Nano-Structures of Ministry of Education, School of Physics and Technology, 12390 Wuhan University, Wuhan 430072, China; Wuhan Institute of Quantum Technology, Wuhan 430206, China; Henan Academy of Sciences, Zhengzhou 450046, China

**Keywords:** nanocavity, plasmonic, coupling efficiency, Gaussian beam, impedance match

## Abstract

Plasmonic nanocavities have emerged as a powerful platform for extreme light confinement, enabling transformative applications in single-molecule Raman spectroscopy, ultra-sensitive sensing, strong light–matter interactions, etc. By harnessing localized surface plasmons, these nanostructures support unprecedented field enhancement, exceeding 1,000-fold in the sub-nanometer gap. However, a fundamental trade-off exists between deep sub-wavelength field localization and its efficient coupling to free-space light, limiting their practical performance. Here, we show that by balancing the electric and magnetic resonance, more than 55 % of a focused Gaussian beam can be fueled into a nanocube-on-mirror nanocavity. With few concentric gratings, the coupling efficiency can even go up to >95 % at optimal conditions. This design can work at both visible and telecommunication wavelengths and show robust tolerance to fabrication imperfections. Our work indicates that the long-standing conflict between deep field localization and efficient external coupling in plasmonic systems can be resolved by multiscale structure design, promising the use of a single metal nanoparticle for advanced nanophotonic or optoelectronic devices.

## 1 Introduction

Plasmonic nanocavities enable extremely localized field enhancement at sharp tips [1] or within nanoscale gaps [2], [3], providing revolutionary platforms for enhanced light–matter interactions [4], [5], [6], [7], such as single-molecule spectroscopy [8], [9], ultra-sensitive sensing [10], [11], [12], and detection [13]. This exceptional capability stems from the collective oscillations of conduction electrons at metal–dielectric interfaces, called localized surface plasmons (LSPs). They exhibit absorption and scattering cross-sections exceeding the physical dimensions of the metal nanostructures, enabling the plasmonic nanocavities a platform for studying cavity quantum electrodynamics at room temperature [6]. As the characteristic length, like the diameter of a nanosphere or the gap distance between adjacent metal nanostructures decreases, plasmonic nanocavities progressively approach the theoretical limits of field confinement [14], [15], [16]. However, the coupling efficiency between these systems and the free-space light also scales approximately as (*l*/*λ*)^2^ [17], where *l* represents the effective current dipole length of the plasmonic system and *λ* is the wavelength of light. This implies a fundamental trade-off between the capacity of light localization and the coupling efficiency to external radiation. That is to say, while reducing the physical length like the nanocavity gap enhances the local fields, it simultaneously decreases *l* to maintain the resonance wavelength, thereby diminishing the coupling efficiency. Take the widely used nanocube-on-mirror (NCOM) as an example, the resonant wavelength remains the same if the gap distance and the edge length of the nanocube are reduced simultaneously [18]. This NCOM nanocavity, taking advantage of its controllable fabrication and superior near or far field properties, has been extensively utilized for strong plasmon–exciton coupling [18], [19], [20], [21], [22], enhanced Raman scattering [16], [23], [24], [25], and fluorescence enhancement [4], [26], [27], [28], [29].

Yet, the scattering or absorption cross-section of an NCOM is typically on the order of 10^−3^ ∼ 10^−1^ μm^2^ [18], quite smaller than the diffraction-limited beam size of light. For instance, for a single nanocube with the edge length of 60 nm and 5.8 nm gap distance, the absorption cross-section of NCOM is 0.11 μm^2^ at its resonance wavelength of 740 nm, only accounting for 26 % of the ideally focus spot area of ∼*πλ* ^2^/4. This restricts the coupling efficiency between free-space photons and the nanocavities – the experimentally measured absorption has a peak value around 20–30 %, even excited by a tightly focused Gaussian beam under a high numerical aperture object [30], [31]. To achieve perfect absorption of light, a dense assemble of NCOM up to a surface coverage of 20 % or larger is required [32], [33], [34], [35], [36]. On the other hand, the use of concentric ring gratings enables the conversion of free-space photons into focused surface plasmon polaritons (SPPs) [37], [38], [39], generating field enhancement at the central position [40]. This effect subsequently enhances the electric field intensity in the localized environment surrounding the nanoantenna [41], [42], [43], [44], [45], [46], [47], [48] and fluorescent molecules (or quantum dots) [49], [50], [51], [52], [53] positioned at the center of the ring, generating directional emission enhancement, thereby improving coupling efficiency. By using the concept of the metasurface, light can also be efficiently fueled into focused SPPs where a nanocavity is located [54]. Although such metasurface design can even achieve near-unity conservation of light to surface waves, the total efficiency of light fueled into a single nanocavity is still limited to less than 60 % [54]. In addition, their large sizes and difficulty during large-area fabrication will limit practical applications. The concept of perfect absorber or coherent perfect absorption is applied only for an array of nanostructures illuminated by a plane wave, and the optimization of coupling efficiency between a single plasmonic nanocavity with a focused light beam in free space remains elusive. Also, intermode interference such as the Kerker condition in light scattering by a single nanoparticle has not been used to improve such coupling efficiency.

Here, we realize near-unity coupling of a focused beam to an NCOM nanocavity via a cascaded optimization procedure. First, the absorption efficiency of a single NCOM is maximized to larger than 55 % at the ‘perfect absorption’ conditions by balancing the electric and magnetic response of the system. Second, the emission pattern of the NCOM is collimated by concentric circular gratings to improve the mode profile matching with the focused beam. Numerical results demonstrate that the maximum coupling efficiencies can reach 94.5 % and 95.1 % at visible and telecommunication wavelengths, respectively, and maintains high tolerance to fabrication imperfections. This result indicates that the long-standing trade-off between deep field localization and efficient external coupling for plasmonic nanocavities can be mitigated via structure design. The near-unity fueling light into a single nanocavity can renew the long-standing interest in using a single plasmonic nanoparticle for ultra-broadband photodetectors or Purcell-enhanced quantum emitters.

## 2 Methods and structural parameters

**Figure 1:**
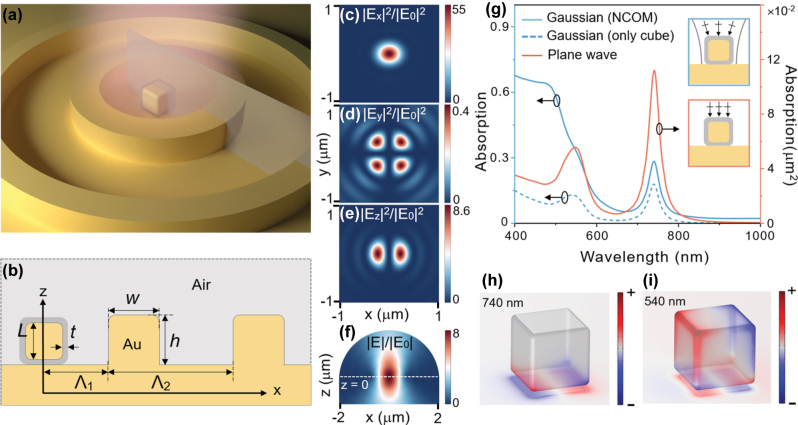
The configuration of g-NCOM and the plasmonic properties of NCOM. (a) Schematic of the designed geometry, consisting of a silica-encapsulated gold nanocube on a gold film, surrounded by the concentric ring grating. (b) Cross-section of the structure with geometrical parameters defined. (c–e) The *x*-, *y-*, and *z*-component of electric field intensity profiles of the focused *x*-polarized Gaussian beam at the focal plane. (f) Electric field profile in the *xz*-plane of the same Gaussian beam, focused from the boundary of a hemispherical computation domain. The wavelength used in (c–f) is *λ* = 740 nm. The white dashed line in (f) indicates the position of *z* = 0 (45 nm below the surface of gold film). (g) Comparing the absorption efficiency under focused Gaussian beam excitation (blue, left axis) and the absorption cross-section under plane wave excitation (orange, right axis) for an NCOM without grating. The heat is integrated over both the nanocube and the film (solid) and only the nanocube part (dashed). The insets show the schematic of a Gaussian beam and plane wave illumination. (h, i) Charge distributions of the NCOM at resonance wavelengths of 740 nm and 540 nm.

The simulated geometry consists of a silica-coated gold nanocube (AuNC) on the gold film, with or without a concentric gold ring grating, illuminated by a linearly polarized focused Gaussian beam (Figure 1a). Numerical simulations of the NCOM or grating-coupled NCOM (g-NCOM) system are performed using a finite element method (FEM) package (COMSOL Multiphysics). In the simulation, we choose two target wavelengths of 740 and 1,550 nm to showcase the potential applications in visible and telecommunication regions. The focused Gaussian beam is theoretically generated by focusing a linearly polarized paraxial Gaussian field via a spherical lens [55]. The numerical aperture of the spherical lens is modeled by the beam radius *r* of the paraxial beam illuminating a hemi-spherical surface. The amplitude of the electric (or magnetic) field of the paraxial beam is denoted as *E*_0_ (or *H*_0_) at the center (maximum) of the beam. The focal lengths of the spherical microlens are approximately 2.0 and 4.5 μm corresponding to the operational wavelengths of 740 and 1,550 nm, respectively. The grating surrounding the AuNC is used to couple incident photons into SPPs, which interferes and generate anti-node at the location of AuNC. Therefore, the grating period Λ_2_ is 2*π*/*β*_spp_ [51], where *β*_spp_ is the propagation constant of the SPPs. For the target wavelengths, it yields Λ_2_ = 720 nm (for 740 nm) and 1,543 nm (for 1,550 nm). The other geometric parameters of g-NCOM are defined as follows. *L* is the edge length of AuNC; *t* is the thickness of the SiO_2_ coating layer around the AuNC; Λ_1_ is the inner radius of the grating wall; *w* is the width of individual grating teeth; and *h* is the height of the grating.

Under the incidence of a focused Gaussian beam, the incident $\left(P_{\text{inc}}\right)$ , transmitted $\left(P_{\text{trans}}\right)$ , reflected $\left(P_{\text{ref}}\right)$ , and absorbed optical power $\left(P_{\text{abs}}\right)$ were calculated to define the absorption efficiency (*η*), satisfying *P*_inc_ = *P*_trans_ + *P*_ref_ + *P*_abs_. Given the sufficient thickness of the bottom gold film, the transmitted power can be negligible (i.e., *P*_trans_ = 0). A perfectly matched layer is placed beneath the structure to fully absorb the incident light, ensuring no reflection occurs during the light propagation. The absorbed power *P*_abs_ was calculated by integrating the Ohmic heating inside the AuNC, gratings, and the entire gold film. The incident power *P*_inc_ was obtained by integrating the Poynting vector over the incident plane in a separate benchmark simulation where the entire computational domain was set to an air background with permittivity of 1. Thus, the absorption efficiency can be defined as *η* = *P*_abs_/*P*_inc_. The mesh size is refined until the convergence. The refractive index of gold is adopted from Johnson and Christy’s results [56], while that of silica is set to 1.5.

## 3 Results and discussion

Figure 1c–e shows the electric field components of a focused Gaussian beam in the focal plane, consistent with the text book results [55]. The *x*-component of the electric field intensity is focused into a beam with a radius of approximately *λ*/2 [55]. |*E*_*x*_|^2^ reaches up to 55 times compared to the amplitude |*E*_0_|^2^ of the *x*-polarized incident paraxial beam (Figure 1c), dominating the other two components. As shown in Figure 1d, the *y*-component exhibits a clear four-lobe pattern, i.e., the intensity maxima (0.4 times) positioned at 90° intervals in the focal plane. The *z*-component displays a characteristic two-lobe distribution (Figure 1e), with two primary intensity lobes appearing in anti-phase configuration on opposite sides of a nodal line. Figure 1f shows the electric field distribution of the focused Gaussian beam in the *xz*-plane. As expected, the beam converges at the focal plane diverse quickly, depending on the numerical aperture of the spherical lens.

To identify the eigenmodes of the NCOM nanocavity and demonstrate its absorption capability of incident light, the total absorption efficiency under focused beam excitation and the absorption cross-section under plane-wave excitation were calculated and compared. As shown in Figure 1g, for an NCOM with an AuNC of *L* = 60 nm and a rounding radius of 5 nm at all the edges and corners, two main resonance peaks appear at 740 nm and 540 nm for both focused Gaussian beam and plane wave. The spectrum of absorption cross-section under plane-wave excitation corresponds well with the absorption efficiency under Gaussian beam excitation at the plasmon resonances, as the resonance condition is determined by the system’s intrinsic eigenfrequencies [57]. Specifically, the absorption cross-section of the NCOM at 740 nm is 0.112 μm^2^, still smaller than the diffraction-limited beam size of ∼*πλ* ^2^/4 (0.43 μm^2^). This substantial mismatch severely limits the coupling efficiency between a single NCOM nanocavity and external optical fields. Under the excitation of a focused Gaussian beam, the absorption efficiency of the NCOM nanocavity at 740 nm is only 28.5 %, indicating that it cannot achieve perfect coupling with external optical fields. The charge distribution of the plasmon mode at 740 nm is shown in Figure 1h. The optical field is primarily localized in the nanogap between AuNC and gold film, and the opposite charges are distributed on two sides of AuNC’s bottom surface, while the opposite charge is accumulated on the gold film. This mode is identified as a magnetic dipole mode, since it forms an effective current loop around the gap [58]. The charge distribution of the plasmon mode at 540 nm reveals a quadrupole pattern on the surface of AuNC, with gold film showing a complementary dipolar distribution opposing the AuNC’s bottom charges (Figure 1i). It can be identified as a quadrupole–dipole coupling mode and is not of primary interest in this study. At both resonant peaks, the absorption inside the AuNC contributes predominantly to the total absorption of the system (Figure 1g). However, in the short wavelength region, the contribution of absorption inside AuNC to the total absorption becomes weaker. This suggests that the energy in this spectral regime is no longer strongly localized within the nanocavity but instead distributed across the metal film as well.

**Figure 2:**
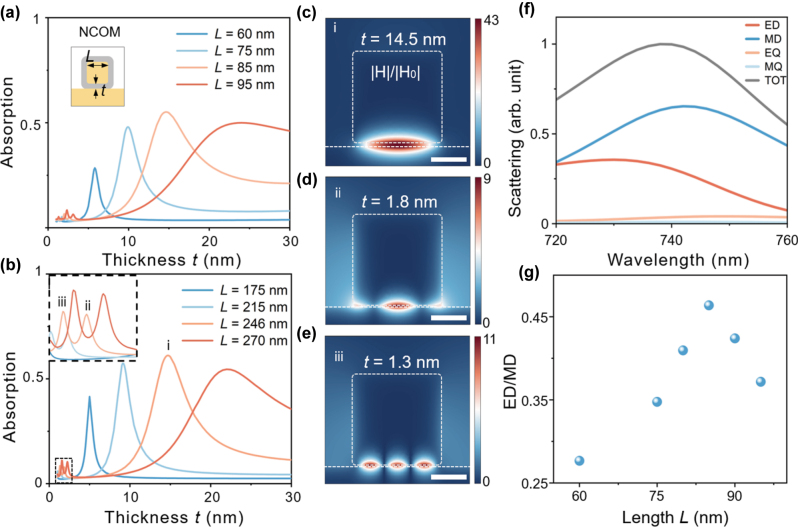
Absorption efficiency with varied silica thickness *t* for NCOM under excitation of 740 nm (a) and 1,550 nm (b) focused Gaussian beam. The inset in (b) shows an enlarged image marked by the dashed lines. Three peaks are labeled as i–iii for *L* = 246 nm. (c–e) Magnetic field distributions of NCOM corresponding to three peaks labeled in (b). The scale bars are 100 nm. (f) Total scattering cross-section and contribution of each multipole. (e) The ratio of MD to ED contributions for different particle sizes.

The structural parameters of the NCOM system, i.e., silica thickness *t* and AuNC size *L*, were optimized to maximize the absorption efficiency in the first optimization step. Except for relatively small *t* where the higher order mode appears, absorption reaches the maximum at the magnetic dipole resonance (Figure 2a and b) at the two designated wavelengths. For larger *L*, the corresponding optimized *t* becomes larger to maintain the magnetic resonance at same wavelength [18]. Interestingly, the NCOM system exhibits a size-dependent absorption efficiency, reaching 28.5 %, 48 %, 55 %, and 50 % for AuNC with *L* = 60, 75, 85, and 95 nm, corresponding to spacer thickness *t* = 5.8, 9.9, 14.5, and 24 nm for 740 nm excitation. For the telecommunication wavelength at 1,550 nm, there is a similar size-dependence behavior and the maximum absorption efficiencies increase slightly to 41.5 %, 57.4 %, 60.8 %, and 54.5 % for *L* = 175, 215, 246, and 270 nm and *t* = 5, 9.1, 14.5, and 22 nm (Figure 2b). The maximum absorption occurs when the structural parameters are close to the ‘perfect absorption’ condition for a nanopatch array [33], [59] or for random deposited NCOM [32]. To examine if the shape of the AuNC affects, we changed the AuNC from a perfect nanocube to a cuboid in the vicinity of the optimized conditions. The results revealed that a cuboid with dimensions *Lx* = 90 nm, *Ly* = 60 nm, and *Lz* = 90 nm achieved a slightly larger absorption efficiency of 56 % at its resonant wavelength. Nevertheless, this represents only a marginal improvement, demonstrating the robustness of optimal absorption efficiency against dimensional variations. Thus, we retained the use of a perfect nanocube in the following optimization due to its near-optimal performance and practical synthesis.

For small silica thickness *t*, there are small absorption peaks corresponding to high-order gap modes. The magnetic field of the three modes for *L* = 246 nm are shown in Figure 2c–e. A pronounced magnetic enhancement is observed in the gap between the AuNC and the gold film, reaching 43 times for the magnetic mode and around 10 times for the two higher-order modes. The weaker field enhancement is due to less efficient to be excited and the standing-wave characteristic suggests that they can be interpreted well within the Fabry–Perot resonances of the metal–insulator–metal waveguide [57], [60].

The central idea of maximizing the absorption efficiency for a single plasmonic nanocavity is to decouple the electric and magnetic response of the system, so that impedance matched to free space can be achieved [61]. To investigate the amplitude ratio between the electric dipole (ED) and magnetic dipole (MD) modes at the same resonant wavelength for particles of different sizes, we performed a multipole expansion to account for their contribution to the scattering cross-section of the NCOM [62]. Multipole moments including ED, MD, electric quadrupole (EQ), and magnetic quadrupole (MQ) are computed independently through spatial integration of the current density **J**(**r**) within the nanocavity [62]. For instance, the ED and MD moments are given by

(1.1) $$\begin{array}{ll} p_{i} & =\frac{1}{-i\omega }\left\{\int J_{i}\left(r\right)j_{0}\left(kr\right){\text{d}}^{3}r+\frac{k^{2}}{2}\int \left[3\left(r\cdot J\left(r\right)\right)r_{i}\right.\right.\\ & \;\left.\left.-r^{2}J_{i}\left(r\right)\right]\frac{j_{2}\left(kr\right)}{{\left(kr\right)}^{2}}{\text{d}}^{3}r\right\} \end{array}$$

(1.2) $$m_{i}=\frac{3}{2}\int {\left(r\times J\left(r\right)\right)}_{i}\frac{j_{1}\left(kr\right)}{kr}{\text{d}}^{3}r$$

where the subscript *i* denotes *x*, *y*, *z* and *k* is the wavenumber in air. In order to eliminate the influence of propagating surface plasmon polaritons on the localized mode decomposition, integration is carried only in the nanoparticle and a segment of the substrate, typically defined to have a width and length of twice those of the nanoparticle. This truncated domain is not accurate but provides a practical way to compare the amplitude of different multipole moments qualitatively. The scattering spectra are the sum of contributions from the ED, MD, EQ, and MQ (Figure 2f). Subsequently, we plotted the ratio of MD to ED contributions for different particle sizes (Figure 2g). As shown in Figure 2f, near the resonant wavelength, the contributions from the MD and ED dominate, with the magnetic dipole exhibiting a stronger intensity than the electric dipole. As the size of AuNC increases, the MD/ED ratio first rises and then declines, reaching its peak at *L* = 85 nm (Figure 2g). This trend correlates well with the optimal absorption observed in Figure 2a, indicating that these optimal conditions are determined by a closer to the balance of the amplitude of the orthogonal orientated electric dipole and magnetic dipole mode. In other words, the effective electric permittivity and magnetic permittivity become closer to each other so that the impedance of NCOM at the resonance matches that of the free space [58]. From another point of view, an orthogonal orientated electric and magnetic dipole with equal amplitude will lead to a unidirectional emission perpendicular to the metal film, which optimized the mode profile matching with the incident light. Figure 2g also indicates that due to the limit of NCOM geometry to tune the electric and magnetic response independence, the amplitude of ED and MD is still away from the perfect matching so that further optimization is still required.

**Figure 3:**
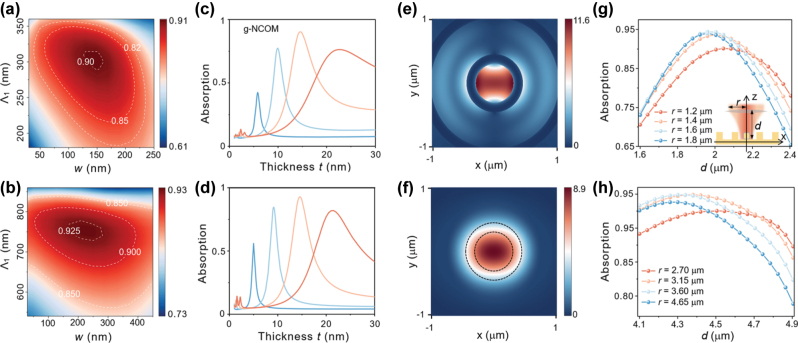
Mapping of absorption efficiency with the varied width *w* and inner radius Λ_1_ under excitation of 740 nm (a) and 1,550 nm (b). Absorption efficiency with varied silica thickness *t* for g-NCOM under excitation of 740 nm (c) and 1,550 nm (d) focused Gaussian beam. Electric field amplitude at a plane 55 nm above the metal surface with (e) and without (f) the grating under 740 nm excitation. The black dashed lines in (f) correspond to the position of grating in (e). Absorption efficiency of the g-NCOM system as a function of *d* with different *r* under excitation of 740 nm (g) and excitation of 1,550 nm (h), and the inset in (g) shows the definition of *d* and *r*.

To optimize the grating parameters of the g-NCOM system, the mapping of absorption efficiency against the varied grating width (*w*) and the inner radius (Λ_1_) at 740 nm and 1,550 nm excitation was calculated and shown in Figure 3a and b. It reaches 90.3 % at Λ_1_ = 300 nm and *w* = 150 nm for 740 nm excitation (Figure 3a), and 92.8 % at Λ_1_ = 760 nm and *w* = 210 nm for 1,550 nm excitation (Figure 3b). Compared to bare NCOM without the grating, the maximum electric field enhancement factor is 1.28 (740 nm) and 1.27 (1,550 nm) times for g-NCOM at the optimized grating parameters (more details in Supplementary Note 1). Such moderate increment of the local electric field indicates that the main effect of the grating is to optimize the matching between the focused Gaussian beam and the g-NCOM system’s radiation pattern at these parameters. The g-NCOM system demonstrates remarkable robustness against grating parameter variations. As shown in Figure 3a, the absorption efficiency of the system maintains above 82 % across wide parameter ranges, tolerating ±50 nm variations around the optimal inner radius (Λ_1_ = 300 nm) and grating width from 100 to 200 nm. Similarly, the absorption efficiency in Figure 3b remains more than 85 % even with significant parameter variations, including the inner radius from 600 to 800 nm and width between 100 and 400 nm. This robust performance is achieved by keeping Λ_2_ (grating period) constant during optimization, ensuring the consistent excitation of focused SPPs despite geometric variations in other parameters. These results highlight the system’s tunability across wavelengths and its compatibility with practical fabrication constraints. Remarkably, the optimal absorption efficiency of g-NCOM reaches 42.5 %, 77.4 %, 90.3 %, and 75.3 % for *L* = 60, 75, 85 and 95 nm (Figure 3c), showing an impressive improvement compared to the bare NCOM (Figure 2a). Importantly, the introduction of the grating does not significantly alter the resonant wavelength and lineshape of the NCOM except for enhancing its peak intensity (more details in Supplementary Note 2), indicating that the grating predominantly collimates the radiation pattern. The simulations confirm that the g-NCOM maintains absorption efficiency even for AuNCs beyond the optimal size range. For example, it maintains >75 % of absorption efficiency across a ±10 % size variation range of optimal AuNC parameters, highlighting its robustness against structural imperfections. For a cuboid with unequal length, width, and height (*Lx* = 90 nm, *Ly* = 60 nm, and *Lz* = 90 nm), the maximum absorption efficiency is slightly less (89.3 %). Under 1,550 nm excitation, the absorption efficiency of g-NCOM is enhanced to 56 %, 84.6 %, 92.8 %, and 80.9 % (Figure 3d). The absorption enhancement by grating stems from two synergistic mechanisms: (i) The concentric ring grating architecture facilitates efficient conversion of free-space photons into focused SPPs [33], [34], [35], creating significant field enhancement at the central position [36]. This is evidenced by the electric field distributions on the gold film surface under focused Gaussian beam illumination without AuNC (Figure 3e and f), which show that the maximum electric field amplitude with the grating is approximately 30 % higher than that without the grating. This plasmonic focusing effect directly amplifies the magnetic mode intensity within the NPOM nanocavity, consequently enhancing the system’s overall optical absorption rate. (ii) The gratings transform the originally isotropic ellipsoidal radiation pattern [17] into a sharp needle-like distribution along the normal direction of the metallic film [47], [51]. Such a highly directional main lobe has superior spatial mode matching with the incident Gaussian beam (more details in Supplementary Note 3). Even under a focused beam irradiation at a tilt angle of 25°, the absorption efficiency of the g-NCOM system can remain above 65 %. Both mechanisms operate complementarily but are also correlated. The former boosts nanocavity–photon coupling through intensifying the background optical field surrounding the AuNC. The interference of the SPP intensifies the near-field at the AuNC and its emission in the normal direction but suppresses the radiation to all other directions, leading to a collimated radiation pattern.

To investigate the influence of both the numerical aperture of the spherical lens and the focal plane position on the absorption efficiency, we varied the distance *d* between the spherical lens and the surface of the gold film, and calculated the absorption efficiency of g-NCOM for incident paraxial Gaussian beam of different waist radius *r* in Figure 3g and h. A schematic of the excitation geometry is shown in Figure 3g, where a paraxial Gaussian beam is focused onto the g-NCOM system by a spherical lens. The absorption efficiencies in Figure 3g and h reach maximum when *d* is close to the focal length of the spherical lens. Remarkably, the maximum absorption efficiency reaches 94.5 % and 95.1 % at 740 nm and 1,550 nm excitation, respectively. For a spherical lens with larger numerical apertures, i.e., incident paraxial beam of larger radius *r*, the focus depth is smaller and so the distance-dependent absorption efficiency varies more rapidly. In addition, the maximum absorption efficiency increases but then declines due to the imperfect match of the radiation pattern. This further confirms that the role of grating is to collimate the radiation pattern of g-NCOM to match with the incident Gaussian profile. For the incident light at 740 nm, the absorption efficiency of the g-NCOM remains above 65 % when the distance *d* is varied by less than 20 % with respect to the focus length, i.e., |*d* − *f*|/*f* < 20 %, where *f* denotes the focal length. It remains higher than that of an NCOM without a grating structure. In the case of 1,550 nm, the absorption efficiency of the g-NCOM remains above 80 % when |*d* − *f*| < 400 nm. This shows a good tolerance to variations in incident light parameters.

**Figure 4:**
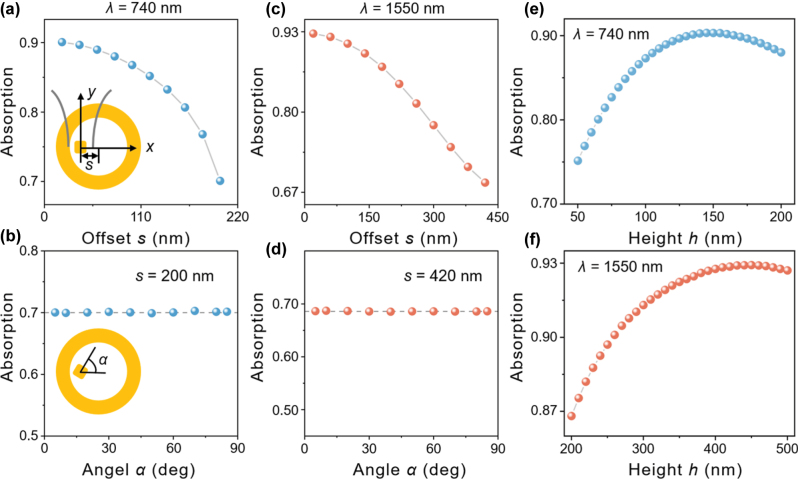
Absorption efficiency as a function of the offset distance *s* (a, c) and the rotation angle *α* with *s* = 200 nm (b) and *s* = 420 nm (d), where (a, b) and (c, d) are under 740 and 1,550 nm excitation, respectively. The insets in (a, b) show the definition of *s* and *α*. Absorption efficiency against the grating height with the excitation of 740 nm (e) and 1,550 nm (f).

To further evaluate the robustness of our system, we examined additional parameters to account for practical implementation scenarios. Since the silica-coated nanoparticles are randomly dispersed on the metal film in most real cases, their positions cannot be precisely aligned with the grating center. This necessitates that the absorption efficiency remains relatively insensitive to the nanoparticle position. To study the influence of the relative position of AuNC against the grating for the absorption efficiency, the offset distance *s* and the rotation angle *α* were further considered while keeping the focused Gaussian beam aligned with AuNC. We simulated the variation in absorption efficiency as the AuNC was displaced along the *x*-axis from the grating center (Figure 4a and c). The monotonic decline in absorption efficiency was observed with increasing *s*, originating from the nanoparticle’s displacement away from the maximum field intensity region at the focal point of the confined SPP mode. The result demonstrates that the g-NCOM system maintains significantly higher absorption efficiency compared to the grating-free configuration, even when the nanoparticle nearly approaches the grating (*s* = 200 nm in Figure 4a and *s* = 420 nm in Figure 4c). This robust performance under extreme proximity conditions demonstrates the g-NCOM system possesses superior absorption characteristics compared to the bare NCOM. The polarization dependence of the nanoparticles is systematically investigated by fixing their positions at *s* = 200 nm for the 740 nm excitation and *s* = 420 nm for the 1,550 nm excitation. When the AuNC is rotated from *α* = 5°–85°, the corresponding absorption efficiency always remains a constant for two wavelength excitations (Figure 4b and d). Remarkably, the absorption efficiency exhibits exceptional stability against rotation of nanoparticle, with variations remaining below 0.5 % across the entire angular range in both configurations. It indicates that the g-NCOM system’s performance depends primarily on the nanoparticle’s distance from the grating center and is insensitive to its rotational orientation. This rotational invariance occurs because (i) the NCOM supports degenerated magnetic dipole mode due to the equal length and width of the AuNC, and (ii) the focused Gaussian beam’s main field distribution at the focal plane exhibits near-perfect cylindrical symmetry with a spot diameter significantly exceeding the nanoparticle’s dimensions, ensuring uniform excitation regardless of in-plane rotation angles. Such polarization insensitivity substantially improves the experimental feasibility of our design by eliminating complex polarization alignment procedures while maintaining stable performance under natural unpolarized light conditions. This is a critical advantage for realistic applications ranging from solar energy harvesting, light detector, to biomedical sensing.

Finally, we investigated the effect of grating height *h* on the absorption efficiency of the g-NCOM system. Figure 4e demonstrates that the g-NCOM system maintains exceptional absorption stability (>80 %) across the grating height range of 75–200 nm, reaching a maximum efficiency of 90.3 % at the optimal height of 145 nm. Remarkably, this performance is further improved in the extended range of 200–500 nm for 1,550 nm excitation, where the absorption efficiency remains above 85 % and the maximum reaching 92.9 % when the grating height is 450 nm (Figure 4f). The results reveal that the g-NCOM system is largely insensitive to variations in grating height, demonstrating the robustness against positional misalignment of AuNC, random polarization of incident light, and dimensional tolerances of gratings. This makes the g-NCOM system highly suitable for practical applications where the positioning of AuNC, accurate nanofabrication, and exact excitation conditions cannot be precisely guaranteed.

As required by the reciprocity, an efficient in-coupling of light into a deep subwavelength plasmonic nanocavity can benefit for the reverse process like single photon emission, which is one of the key elements for scalable photonic quantum computing and quantum communication and detection. Plasmonic nanocavities have been shown to significantly enhance fluorescence rates [27], [63], [64], but they are often accompanied by the presence of quenching [65] that reduces the quantum yield of the emitter. To estimate the performance of g-NCOM for single photon emission, we compared (i) a typical NCOM with an edge length of 60 nm and a gap distance of 5.8 nm, (ii) an optimized NCOM (edge length 85 nm, gap distance 14.5 nm), and (iii) optimized NCOM with concentric gratings. Comparing the optimized NCOM with the typical one, the local density of state is reduced from 3.8 × 10^4^ to 1.8 × 10^3^ due to the large gap distance, and the quantum yield increases from 10.8 % to 42.1 %. Comparing case iii with ii, the presence of grating changes the quantum yield slightly from 42.1 % to 45.3 %, while the radiation power (collected with a numerical aperture of 0.9) is enhanced by 43.8 %. The results show that the optimized in-coupling conditions offer a promising strategy to simultaneously enhance directionality and suppress quenching for an emitter, which is essential for the end-to-end single-photon source efficiency [66].

## 4 Conclusions

In summary, by implementing focused Gaussian beam into electromagnetic modeling of the widely used plasmonic nanocavities, we can optimize the coupling efficiency of a single NCOM nanocavity with external excitation. The results show that by introducing concentric metal gratings around the NCOM nanocavity, more than 90 % of the excitation light can be fueled into a single nanocavity at designed wavelengths while maintaining fabrication robustness. Such a simple design can avoid the long-standing trade-off between field confinement and external coupling efficiency at a moderate price of fabrication cost, compared with a more elegant metasurface design. It can advance the design for light collection or light emission, such as broadband single-photon detectors or ultrabright single-photon sources.

## Supplementary Material

Supplementary Material Details
