# Supplementary material for: Near-unity fueling light into a single plasmonic nanocavity
Source: Nanophotonics. 2025 Nov 6;14(27):5069–79. doi: 10.1515/nanoph-2025-0331 (PMC12717919; doi:10.1515/nanoph-2025-0331)
Supplement: Supplementary file 1 — Supplementary Material Details [file j_nanoph-2025-0331_suppl_001.pdf]

Supplementary Note 1

We have calculated and added the electric field distribution of NCOM and the maximum electric field amplitude of g-NCOM with the varied parameters of the grating in Fig. S1. The analysis reveals a positive correlation between the absorption efficiency and the field enhancement factor. The patterns in the enhancement map closely mirror those in the absorption maps (Fig. 3a-b), and the peak enhancement is achieved precisely at the same optimal structural parameters that maximize absorption. The results confirm that optimizing the absorption efficiency simultaneously optimizes the local field intensity, demonstrating that the g-NCOM is also effective to enhance both the excitation and emission, benefiting processes like surface-enhanced fluorescence, SERS, etc.

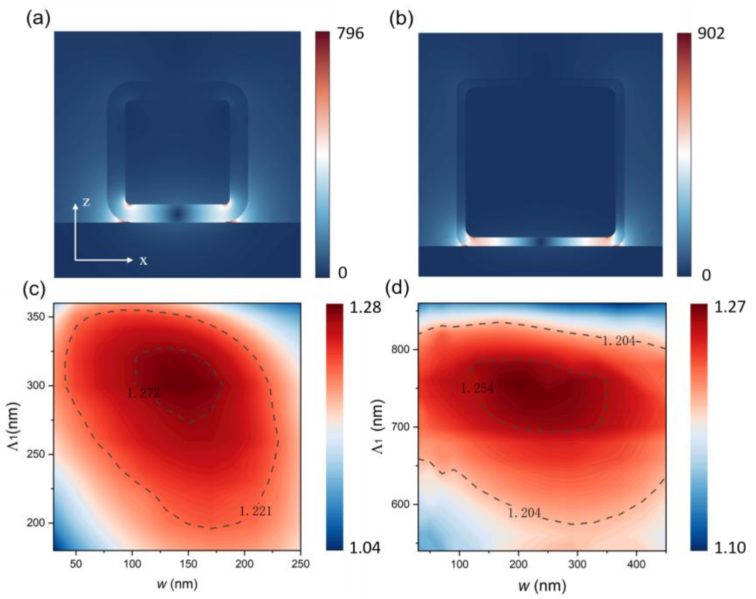

Fig S1. Electric field distribution in the xz plane of NCOM at 740 nm (a) and 1550 nm (b) excitation. Field enhancement factor of g-NCOM hybrid with the varied grating parameters under 740 nm (c) and 1550 nm (d) excitation. The field enhancement factor is defined as the maximum electric field amplitude ($|E|$) of the g-NCOM in the xz-plane divided by the maximum electric field amplitude of the NCOM under corresponding conditions.

Supplementary Note 2

we have added two figures that summarize the resonant wavelengths corresponding to the grating widths and inner radius of the grating presented in Fig. 3a-b. These data show that the resonant wavelength remains unchanged at the optimal structural

parameters (w = 150 nm, Λ_1 = 300 nm at 740 nm and w = 760nm, Λ_1 = 210 nm at 1550 nm). While minor shifts in resonance (<1.5%) are observed for some non-optimal dimensions, no spectral shift occurs within the region near the optimum. We therefore conclude that these slight deviations have negligible impact on our main conclusions. To investigate whether the grating structure alters the line shape of the absorption spectrum, we compared the absorption spectra of the g-NCOM and NCOM systems. The resonant peak positions remained unchanged in both structures at around 740 nm and 1550 nm. The full width at half maximum (FWHM) of the absorption peak at 740 nm was approximately 50 nm in both the g-NCOM and NCOM structures. At 1550 nm, the FWHM was about 130 nm for NCOM and 115 nm for g-NCOM. These results indicate that the grating does not modify the spectral profile in terms of resonance position or overall line shape, but primarily enhances the peak absorption intensity. Therefore, we conclude that the grating effectively increases the absorption efficiency without substantially altering the LSPR of the NCOM.

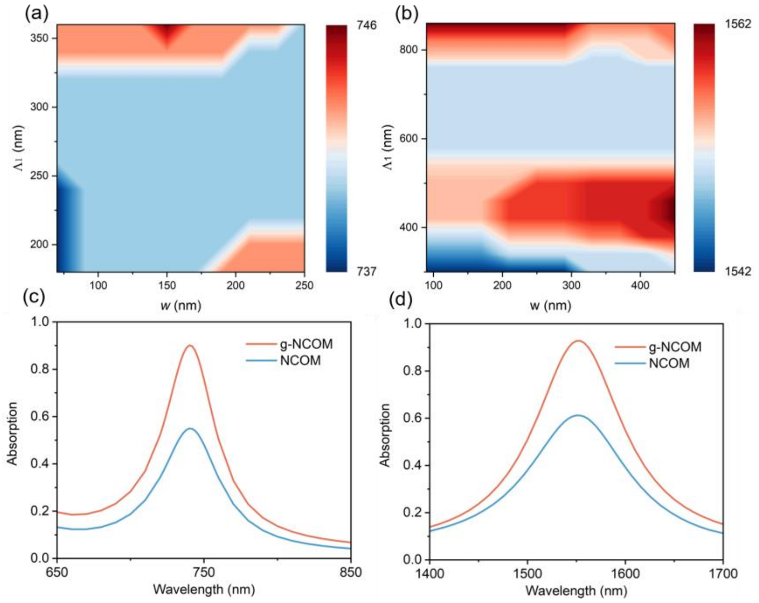

Fig S2. Varied resonant wavelength of g-NCOM relative to the inner radius of the grating wall (Λ_1) and the width of individual grating teeth (w) under the 740 nm (a) and 1550 nm (b) excitation. Absorption spectrum of the NCOM and g-NCOM systems under 740 nm (c) and 1550 nm (d) excitation.

Supplementary Note 3

We calculated the absorption efficiency of g-NCOM under the oblique incident angles ranging from 0° to 25° at both 740 nm and 1550 nm in Fig. S3. Due to the limitation of spherical wavefront in our model, larger incident angles could not be modeled. For comparison, we also computed the normalized far-field radiation pattern of the g-NCOM. As the incident angle θ_1 increases, there is a noticeable decrease in absorption efficiency, following the far-field radiation pattern. Nevertheless, even at a tilt angle of 25°, the absorption efficiency remains above 65% for both wavelengths. These results indicate that the g-NCOM maintains reasonably robust performance under oblique incidence.

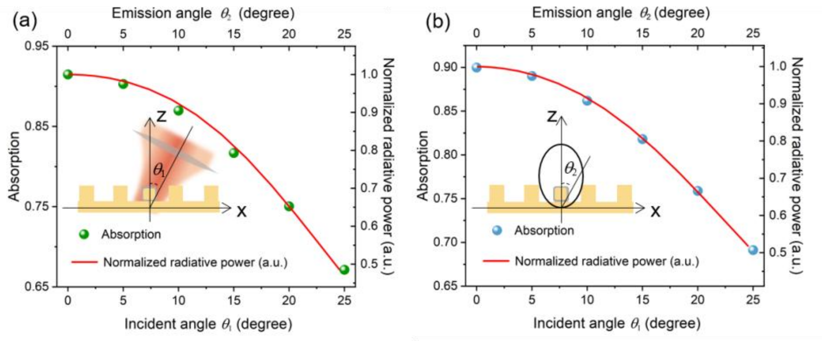

Fig S3. Absorption of the g-NCOM (blue dots) as a function of incident angle θ_1. The normalized far-field radiation power (red line) as functions of emission θ_2 under 740 nm (a) and 1550 nm (b). The inset in (a) and (b) illustrates the definition of incident angle θ_1 and emission angle θ_2.
